# Implementing bedside electronic transfusion checks at Barts Health NHS Trust: A study protocol for evaluating the effectiveness and value for money

**DOI:** 10.1111/tme.70002

**Published:** 2025-08-19

**Authors:** Montasir Ahmed, Laura Green, Iram Bhatti, Catherine Booth, Louise Bowles, Ollie Djurdjevic, Helinor McAleese, Josephine McCullagh, Michael F. Murphy, Florence Oyekan, Nathan Proudlove, Florian Tomini, Yan Feng

**Affiliations:** ^1^ Wolfson Institute of Population Health, Queen Mary University of London London UK; ^2^ Barts Health NHS Trust London UK; ^3^ NHS Blood and Transplant London UK; ^4^ Blizard Institute, Queen Mary University of London London UK; ^5^ Oxford University Hospitals NHS Foundation Trust Oxford UK; ^6^ University of Oxford Oxford UK; ^7^ Alliance Manchester Business School The University of Manchester Manchester UK

**Keywords:** Bedside Electronic Transfusion Checks (BETC), blood transfusion, effectiveness and value for money, study protocol, UK NHS

## Abstract

**Objective:**

To evaluate the benefits of implementing Bedside Electronic Transfusion Checks (BETC) to patients and value for money at four hospitals at Barts Health NHS Trust.

**Background:**

BETC aims to enhance transfusion safety by reducing errors associated with positive patient identification checks for compatibility, blood sample labelling, and blood component administration. There is limited evidence on the potential benefits to patients and healthcare professionals as well as value for money for implementing BETC.

**Methods:**

The BETC implementation at four hospitals adopted a non‐randomised, staggered, multi‐phase strategy. Alongside the implementation, an evaluation study was conducted. The intervention consists of a portable handheld scanning device and a mobile printer used for printing labels that are attached to the compatibility blood bottles and for verifying the patient's details against blood units prior to blood administration. Eligible patients are those who received blood transfusions or had compatibility tests performed during the evaluation period. The outcomes for evaluation include transfusion‐related errors and cost savings from an NHS perspective. Regression‐based time‐series intervention analyses will be applied to evaluate the impacts of BETC implementation.

**Expected Results:**

The three‐year evaluation includes a 12‐month pre‐implementation period (May 2022 to April 2023) and a 24‐month implementation period (May 2023 to April 2025). All staff involved with bedside transfusion were trained on the new system. Data were collected from different transfusion datasets, process mapping dataset, and Health Economics Inventory dataset.

**Discussion:**

Findings from this evaluation study will provide empirical evidence on the effectiveness and value for money of implementing BETC and will support decision‐making for its wider roll‐out in the UK.

## 1 BACKGROUND

Blood transfusion can be a lifesaving treatment used across various clinical settings, such as surgical, medical, and obstetric care.^1^ Transfusion pathway within the hospital is a multistep process intended to provide safe service to patients, considering each critical control step from the laboratory to the patient's bedside to ensure that the right blood is given to the right patient in every time. Errors or omissions in any part of the transfusion pathway can result in the wrong blood being given to the wrong patient, which could lead to ABO‐incompatible transfusions (called ‘Never Event’), with serious clinical consequences for patients.^2^, ^3^, ^4^

National hemovigilance reports have repeatedly demonstrated that human errors occur across the whole transfusion pathway. Errors at the bedside, particularly those involving positive identification of patients (PPI), collection of blood samples for compatibility testing, and the administration of blood, are the most common causes of transfusion errors.^2^, ^5^, ^6^ Therefore, when manual positive identification checks are used at the bedside, national guidelines recommend further safety measures to enhance transfusion safety. These include having two blood samples taken independently of one another to confirm a patient's blood group prior to issuing group‐specific blood components,^7^ and enforcing a zero tolerance for any compatibility testing samples that have not been labelled correctly.^7^, ^8^ Further, pre‐transfusion checks, including PPI, should be performed independently at the patient's bedside by two members of trained staff before blood administration.^9^ Although these measures have improved transfusion safety, they are not fail‐safe.^10^ Moreover, the requirement for second blood samples before transfusion and mandatory second‐clinician verification before administration are associated with potential delay with transfusion and more resources required.

The UK Infected Blood Inquiry exposed critical systemic failures in transfusion safety, which led to severe consequences for patients and their families. It called for stronger safety protocols and technological advancements to prevent future harm.^11^, ^12^ The implementation of electronic bedside checks (BETC) through barcode scanning offers a robust mechanism to enhance transfusion safety.^13^, ^14^, ^15^ By ensuring accurate matching of blood components to the intended patient, the electronic system significantly reduces the risk of transfusion errors,^15^, ^16^, ^17^ thereby improving patient safety and clinical outcomes. The use of BETC for blood sample labelling and for blood administration can reduce the risks of errors at these critical control steps, as well as removing the need for a second compatibility testing sample and for a second nurse to perform PPI checks prior to transfusion.

A few studies^14^, ^15^, ^17^, ^18^ have demonstrated the benefits to patients and hospitals as the results of introducing BETC. However, the evidence of economic benefits to the NHS from implementing BETC is limited. In 2022, Barts Health NHS Trust decided to implement BETC at its four hospitals. Taking advantage of this big investment, an evaluation study was commissioned which aims to assess the effectiveness and value for money of the new electronic bedside checks system. This paper presents the protocol for the evaluation of BETC implementation.

## 2 METHODS AND ANALYSES

The implementation adopted a non‐randomised, staggered, multi‐phase design. Regression‐based time‐series intervention analyses and descriptive analyses will be applied to evaluate the impacts of BETC implementation. Research Ethics Committee approval (REC Reference Nr 22/NW/0138) and Health Research Authority approval (Nr 311 676) were obtained.

### 2.1 Pre‐implementation status

**FIGURE 1:**
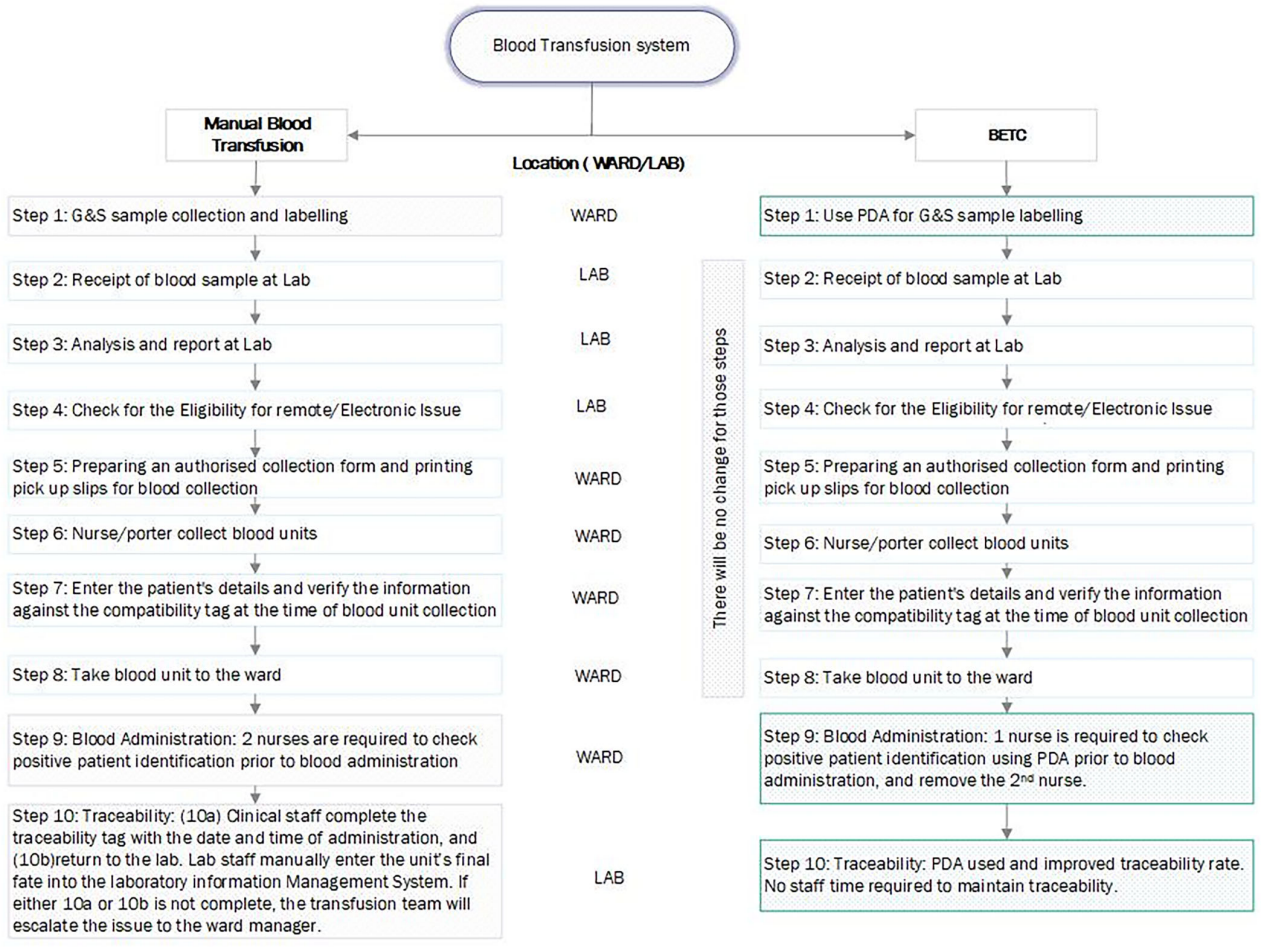
Transfusion process steps under manual and BETC systems. Steps 1, 9 and 10 under the manual system will be replaced by the BETC.

Barts Health NHS Trust comprises four hospitals. Between these, in 2022, ~ 65 000 blood units were administered to ~15 000 patients. The blood transfusion process begins with a clinician's decision to prescribe a transfusion and ends with the administration of the blood unit to the patient. Across the four hospitals, blood transfusion follows a standardised 10‐step procedure, as outlined in Figure 1. Of these, seven steps are currently supported by the electronic system, while step 1 (blood sample collection and labelling), step 9 (blood administration), and step 10 (traceability of the blood unit) are performed manually.

PPI checks must be performed every time for steps 1 and 9. In the manual process, step 1 requires that labelling on the blood sample and the request form is handwritten, where the clinician is required to write the patient's full name, date of birth, hospital number, NHS number, date and time when the sample is taken, and provide his/her signature. For step 9, prior to blood administration, PPI must be performed by two nurses who are required to check that the patient's details on the wristband match those on the blood unit and the compatibility label that is attached to the blood unit. For step 10, clinicians must return the traceability tag attached to the blood unit from the ward to the laboratory, where an individual will enter this information in the transfusion electronic system—this confirms the fate of the unit and is called traceability.

### 2.2 Intervention

The intervention consists of a portable handheld scanning device (or Personal Device Assistance [PDA]) and a mobile printer. For the sample labelling (step 1), the PDA obtains the patient's details electronically via scanning the patient's 2D barcode on the wristband, which is embedded with the patient's details from the patient's electronic record and, in doing so, supports PPI. The PDA then communicates via Bluetooth with the mobile printer to print the label, which is then attached to the blood sample. For blood administration (step 9), the device uses the same 2D barcode to obtain and verify the patient's details against the compatibility label attached to the blood unit. For traceability (step 10), information on the use of blood is transmitted electronically every time a unit is being transfused using the PDA. It should be noted that the BETC can only be used if patients wear wristbands; consequently, these devices will not be implemented in areas where this was not the case (e.g., the outpatient setting for group and screen [G&S] samples).

The routine clinical care of patients receiving transfusions or needing blood samples will not be altered for the purpose of this study.

### 2.3 Theory of change

We anticipate the implementation of BETC will lead to a reduction in the G&S blood sample rejection rate due to avoidable mislabelling errors (step 1), thus reducing the frequency of re‐bleeding patients, resulting in less distress for patients, and a reduction in the workload imposed upon the clinical staff to re‐bleed and the laboratory to process the sample. This will subsequently prevent potential delays in blood provision (and other treatments that depend on transfusion, e.g., surgery) and thus have better outcomes for patients.

BETC can potentially eliminate the need for having two G&S blood sample rules and two clinicians to perform independent pre‐transfusion checks prior to blood administration (step 9) and will improve the traceability process (step 10), as transfusion data will be collected in real time through electronic transmission of the information, providing a full audit trail. We expect all these changes will free up staff time, allowing them to perform other important clinical tasks, and as a result improving the productivity of the four hospitals. Furthermore, we expect the BETC implementation to generate savings from material and consumable use because of the reduction in re‐sampling. Ultimately, the implementation of BETC will prompt clinicians to carry out key steps of blood transfusion correctly, thus avoiding wrong blood being given to the wrong patient, which can have detrimental outcomes for patients.

### 2.4 Implementation phases

The BETC implementation at the four hospitals adopted a non‐randomised, staggered, multi‐phase strategy. The time horizon for the evaluation is 36 months. It includes a 12‐month pre‐implementation period (May 2022 to April 2023) and a 24‐month implementation period (May 2023 to April 2025). The implementation includes three phases:

1. Pre‐Pilot (May 2022 to April 2023): This phase focused on site readiness, addressing all IT requirements for the use of the new system in clinical areas, and preparing training material to accommodate BETC integration.
2. Pilot: The pilot was performed in the Haematology Day Units (HDU) at four hospitals. It allowed the project team to identify and resolve any unforeseen issues that had not been picked up in the pre‐pilot stage. The HDUs were chosen due to the high volume of pre‐planned blood transfusions.
3. Full roll‐out: The main BETC implementation was phased out starting from Hospital 1 (July to November 2023), followed by Hospital 2 (December 2023 to March 2024), Hospital 3 (April to October 2024), and Hospital 4 (November 2024 to April 2025). After the completion of the roll‐out at each hospital, there was a three‐week evaluation period.

The principles of the implementation phases were the same across four hospitals, starting with HDU, followed by clinical wards, and then emergency clinical areas (i.e., the A&E department and theatres). The main part of the implementation included training clinicians on how to use the electronic devices (i.e., PDA and mobile printer) for steps 1 and 9. When 80% of clinical staff were trained in a clinical ward, electronic devices were released to the ward for use. Simulation training videos were developed and made available on the Trust intranet for staff to access. Dummy wristbands and dummy blood units were created for demonstration and to make the staff training reflect real practice as much as possible. After practical training, a competency assessment was obtained from all staff via an interactive PDF document, which included questions to consolidate learning and reinforce key points of the training that had been received.

By the end of the full roll‐out phase, we expect all wards to use the devices for transfusion. Some wards might have started using PDAs for transfusion during the full roll‐out period. We collected data for the PDA uptake at the ward level throughout the 24‐month implementation period.

### 2.5 Study participants

The target population was (a) any patient who has received a blood transfusion for any reason at four hospitals, (b) any patient who had G&S blood samples taken for compatibility testing for any clinical reason during the evaluation period, (c) all clinical staff involved in taking G&S blood samples or administering blood units to patients at four hospitals, and (d) supervisors who delivered the BETC training sessions to clinicians.

### 2.6 Outcome measures

A summary of outcome measures, definitions, and data sources are reported in Table 1. Benefits to patients will be measured by the reduction in transfusion‐related errors, as proxied by the following five outcomes:

1. Number and rate of mislabelled samples*.
2. Number and rate of wrong blood in a tube*.
3. Number and rate of patients who receive wrong blood due to errors related to bedside checks**.
4. Number and rate of patients who are misidentified at the point of transfusion**.
5. Number and rate of wrong blood units collected from the fridge due to errors related to bedside checks.

**TABLE 1:** Outcome measures for the evaluation of BETC implementation.

Outcome measures	Definitions	Data sources
Measures for benefits to patients		
Mislabelled samples	Samples contain incorrect information of the patient	Winpath, BloodTrack Manager
Wrong blood in a tube	A G&S sample contains blood from the wrong patient	Winpath, BloodTrack Manager, national hemovigilance scheme
Patients received wrong blood due to errors related to bedside checks	Administration of an incorrect blood unit to a patient due to failure in verifying patient identity and blood group compatibility	DATIX and national hemovigilance scheme
Patients are misidentified at the point of transfusion	A patient is incorrectly identified before receiving a blood transfusion	DATIX and national hemovigilance scheme
Wrong blood units collected from the fridge due to errors related to bedside checks	Incorrect blood units from storage due to failures in verifying patient details and blood group compatibility	DATIX and national hemovigilance scheme
Measures for clinician time saved		
Mislabelled samples	Time spent on G&S sample collection, labelling, analysis and reporting.	Process mapping
Wrong blood in a tube	Time spent on G&S sample collection, labelling, analysis, reporting.	Process mapping
Manually labelling blood samples	Time spent on writing patients' details for G&S labelling	Process mapping
Manually writing post‐administration documents	Time spent on recording transfusion details after blood administration	Process mapping
Having second nurse for blood administration	Time spent by second nurse for compatibility check	Process mapping
Completing traceability documentations	Time spent on documenting and tracking blood transfusion details	Health Economics Inventory
Spillover effect	Time saved from a whole blood transfusion process after implementing the BETC	Process mapping
Measures for savings from material and consumable use		
Mislabelled samples	Material and consumable costs for G&S sample collection and labelling	Health Economics Inventory
Wrong blood in a tube	Material and consumable costs for G&S sample collection and labelling	Health Economics Inventory

*In cases where outcomes 1 and 2 overlap, the events will be counted only once, under outcome 2.

**Errors that occurred at the point of transfusion, will be counted as outcome 4. Other errors will be counted as outcome 3, that is, ‘errors related to bedside checks’.

Two types of hospital cost savings, as the results of the BETC implementation, will be assessed.

1. Clinician time saved: Time saving will be generated from six sources including time spent on (1) re‐sampling because of mislabelled samples, (2) re‐sampling because of wrong blood in a tube, (3) manually writing blood sample labels, (4) manually writing post‐administration documents, (5) having a second nurse for blood administration, and (6) completing traceability documentations. Also, the implementation of BECT on steps 1, 9, and 10 might generate a spillover effect to other steps, and as a result save clinician time further.
2. Reduction in material and consumable use: Savings will be generated from re‐sampling because of (1) mislabelled samples and (2) having wrong blood in a tube.

### 2.7 Data collection

Data will be collected from three sources: (1) transfusion datasets: electronic laboratory databases Winpath and BloodTrack Manager, hospital‐based incident reporting schemes (or DATIX), and reports submitted to the national hemovigilance scheme, (2) Process mapping dataset, and (3) Health Economics Inventory dataset. The process mapping and Health Economics Inventory datasets were developed specifically for this evaluation study.

#### 2.7.1 Transfusion datasets

The Winpath system includes variables relating to G&S blood sampling and rejection, all blood units issued, and blood units transfused. BloodTrack Manager gathers electronic data regarding the use of PDAs, blood fridges, and BloodTrack kiosks. Incidents relating to non‐conformances or near misses relating to transfusion were collected through DATIX, that is, the Trust's incident reporting and risk management software. Adverse events relating to transfusion were collected through the national hemovigilance scheme. We applied the same data collection strategy across the four hospitals, that is, with the same variables extracted from the four datasets and the same time period applied.

#### 2.7.2 Process mapping dataset

The process mapping dataset provides information about the time spent by clinicians in each step of the transfusion process. A structured data collection approach was used to map the transfusion process across the four hospitals within Barts Health NHS Trust. Clinician‐level time spent data were collected using a standardised audit form, a time‐in‐motion method, and a stopwatch to record process durations systematically. A data collection form was developed to capture the clinician time spent in hospital wards and staff time spent in the laboratories for blood transfusion.

#### 2.7.3 Health Economics Inventory dataset

Two Health Economics Inventory forms were developed for the evaluation study. One form was applied in the staff training survey. Every clinician in this study was invited to complete the Health Economics Inventory form after she/he completed the BETC training. The form collected clinician‐level data on training duration, job title, years of experience in the current job, and feedback on the BETC system. The other form was used to collect data on costs for PDA devices and mobile printers, licence/software costs, and BETC system maintaining costs; material and consumable costs for G&S sample collection and labelling; salaries for BETC implementation staff and proportional salaries for two Transfusion Practitioners on the traceability task.

**FIGURE 2:**
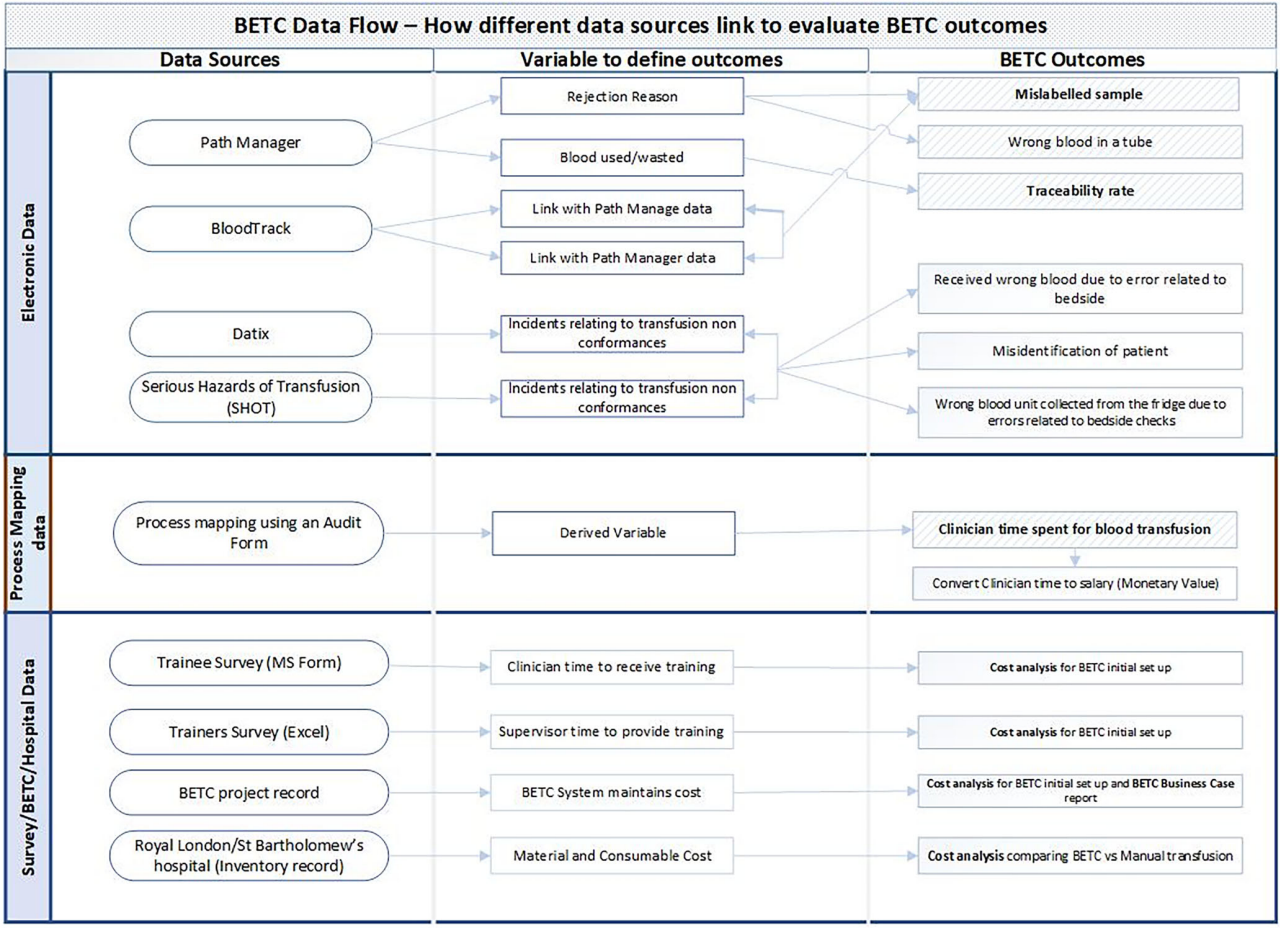
Linking data sources with outcome measures for the evaluation of BETC implementation.

Figure 2 shows the linkage between outcome measures, proxies for outcome measures in various datasets, and the data sources.

### 2.8 Statistical analysis

#### 2.8.1 Benefits to patients within the evaluation period

Regression based time‐series intervention analyses will be applied to evaluate the impact of BETC implementation on the number and rate of mislabelled samples at hospital ward level.^19^, ^20^ The analysis will reflect three key features of the BETC implementation, that is, staggered, adopted gradually, and non‐randomised. We will conduct descriptive analyses to summarise the other four outcome measures on benefits to patients at the hospital and year level.

#### 2.8.2 Cost savings within the evaluation period

Cost savings will be quantified by the monetary value of reduction in (1) clinician time used for completing transfusion‐related tasks and (2) transfusion‐related material and consumable use. We identified seven sources of savings for clinician time and two sources of savings for material and consumable use, as reported in Table 1.

For six (out of seven) sources of clinician time saving associated with BETC implementation, we will estimate the amount of time saved from each source by combining the time saved from one transfusion to the number of relevant transfusions. These will then be converted to monetary value by applying the hourly rate of clinicians.^21^, ^22^ Costs will be presented at 2023/24 level. For traceability, the monetary value of clinician time saved will be estimated by the proportion of salaries for two Transfusion Practitioners in Barts Health NHS Trust for their time spent on supporting the traceability of transfusions under the manual transfusion system. Regarding the two sources of cost savings in material and consumable use, for each source, we will identify and then quantify the material and consumable use items saved from one transfusion, followed by converting the quantities to monetary value by applying the unit cost for each item, and finally to estimate cost savings by combining monetary value from one transfusion to the number of relevant transfusions.

During the 2‐year implementation period, the PDA uptake did not reach 100% for many wards. Therefore, we will report two types of annual cost savings, that is, actual savings and potential savings when the maximum PDA uptake is achieved.

#### 2.8.3 BECT implementation and staff training costs

We will report the actual implementation costs occurred at the Trust and year level. The costs include salary for implementation staff, costs for software licences, PDA hardware maintaining and software support fee, costs for PDA devices and mobile printers. Training costs for clinicians to learn how to use the PDA device will be calculated at clinician level by applying clinicians' hourly rate to the time spent on training sessions.

#### 2.8.4 Benefits to patients beyond the evaluation period

In the UK NHS, transfusion‐related mortality and serious morbidity are rare. Therefore, the immediate impact of BETC implementation on these outcomes is likely to be limited during the implementation period. To assess potential benefits beyond the evaluation timeframe, we will review data from the national hemovigilance scheme, which provides records of all reactions and complications because of ABO‐incompatible transfusions in the UK between 1997 and 2023. We will also conduct a focused literature search to identify published disutility weights for quantifying the impact of adverse events, as a result of mismatched transfusions, on patients' quality‐of‐life. Combining together the national hemovigilance scheme data and disutility weights, we will provide an estimate of the potential long‐term health benefits to patients by reducing preventable transfusion‐related errors across the UK NHS through implementing interventions like the BETC.

We will follow CHEERS guidelines^23^ when reporting the health economic evaluation. All analyses will be conducted using the statistical software STATA 18.^24^

## 3 DISCUSSION

There is considerable pressure worldwide to control the steady increase in the cost of blood transfusion. In transfusion, the costs to deliver the service exceed the costs of the blood products.^25^, ^26^, ^27^ There is limited empirical evidence on interventions that aim to manage the costs of blood transfusion as well as the economic analyses on those interventions.^28^, ^29^

BETC was developed under the strong need for technological advancements to improve transfusion safety. It is expected that the intervention will generate cost savings by freeing up clinicians' time and reducing material and consumable use. Although BETC has been proved to enhance transfusion safety in a few studies,^14^, ^15^, ^17^ the costs aspect of this intervention is unknown. By evaluating the replacement of manual transfusion processes with BETC, this study aims to fill in the literature gap by assessing the benefits of implementing the system to patients and cost savings for the NHS in one of the largest Trusts in the UK. A strength of our study is the detailed level of data collection and, as a result, the large sample size of the linked dataset. The electronic transfusion datasets provide data at the patient level for their transfusion activities. Our study will be the largest health economic evaluation of the electronic transfusion system in the world. Furthermore, we will be able to link the electronic transfusion datasets and the two datasets that were developed specifically for this evaluation study (i.e., process mapping and Health Economics Inventory). The linked dataset provides a unique opportunity for our evaluation to capture a wide range of outcomes as the results of BETC implementation, such as benefits to patients and health professionals and cost savings for hospitals and the NHS. Last but not least, we adopted a micro‐costing approach to collect detailed clinician time spent, resource utilisation, and unit cost data. This methodological choice will enable us to generate accurate estimates of BETC‐related costs and cost savings.

Our evaluation has limitations. The main limitation of the implementation study is the non‐randomised study design. We considered a randomised design at an early stage, but implementing randomisation in real life in transfusion would have been impossible. We opted for a non‐randomised, staggered, multi‐phase implementation design. Our evaluation will address these key features of the implementation. For example, we will apply for a multiple baseline interrupted time series analysis which accounts for the degree of BECT uptake to evaluate the impact of BECT implementation on mislabelled samples. While at the end of the evaluation period all wards within a hospital will have had the electronic devices, the full uptake by clinicians will take months or even longer to achieve. Therefore, we expect hospitals to be at different stages of the BECT uptake during the evaluation period, with Hospital 1 likely to have the highest uptake rate, while Hospital 4 has the lowest. As a result, in this evaluation study, we will not observe the full benefits to patients and cost savings to the NHS. However, based on transfusion activity data, process mapping data, and Health Economics Inventory data, we will estimate the full benefits.

While most hospitals in the UK currently apply the manual blood transfusion system, our evidence from this evaluation will not only generate empirical evidence for its implementation at the Barts Health NHS Trust but also support decision‐making for the technology's wider roll‐out in the UK NHS.

## AUTHOR CONTRIBUTIONS

Laura Green and Yan Feng designed the study, secured funding, and prepared the manuscript. All authors contributed to the delivery of the study and critical review of the manuscript. All authors read and approved the final version of the manuscript.

## FUNDING INFORMATION

This work was funded by Barts Charity and NHS Charities Together (through Barts Charity).

## CONFLICT OF INTEREST STATEMENT

Mike Murphy has received consultancy fees from Haemonetics for speaking at webinars and other events. All other authors declare no conflict of interest.

## Data Availability

Not applicable. This paper is a study protocol which does not involve data or data analysis.
